# Supplementary material for: Using machine learning pipeline to predict entry into the attack zone in football
Source: PLoS One. 2023 Jan 18;18(1):e0265372. doi: 10.1371/journal.pone.0265372 (PMC9847968; doi:10.1371/journal.pone.0265372)
Supplement: Attachment — Submitted filename: Answerletter_PONE-D-22-05975R1.pdf [file pone.0265372.s003.pdf]

PLOS ONE

Response to Reviewers

Thank you for the opportunity to review our manuscript for the PLOS ONE. We appreciate the Reviewer's constructive comments and through reviews.

The content of the manuscript has been revised to address all the Reviewers' concerns. After addressing all the comments and revising the manuscript, we are submitting the revised version of our article for your consideration.

All changes made in the manuscript are highlighted. We considered all comments and notes carefully, and we are sure they represent a significant contribution to the quality of our manuscript.

We thank you for this opportunity. Please let us know if there are any further questions or concerns.

Sincerely

Authors

Title:

Using machine learning pipeline to predict entry into the attack zone in football

---

**Please note that all changes made to the manuscript, legends and figures are highlighted in yellow.

**This document needs to be compiled in Tex Live Version 2021 of Overleaf. The document will not compile in the new version 2022.

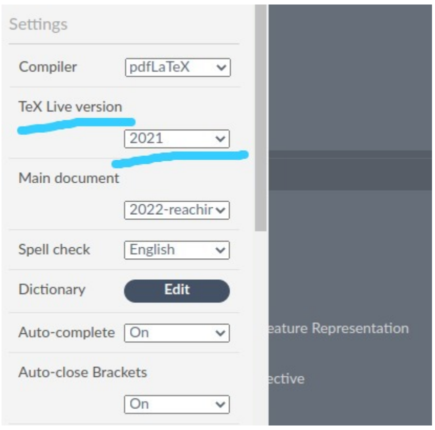

Reviewer 2

1. The motivations or remaining challenges are not so clear, or what kinds of issues or difficulties are this task that is facing. Please give more details and discussion about the key problems solved in this paper, which is largely different from existing works.

R: *The main motivation for this work relies on the lack of effective approaches to predict if a ball possession may lead to a chance of scoring. Therefore, in this paper we aimed to create an effective framework that could lead to insights of how the attacking team may approach the penalty box, i.e., may have more chances of scoring. The main novelty of our work relies on the use of a complex network formulation. Other papers analyzed teams using similar metrics to ours, but for different applications and objectives.*

The main contribution is to show that the use of complex network features to understand and predict the attack behavior is viable. Another contribution relies on the combination of time series representations with convolutional networks, pre-trained with images, to produce effective predictions. To the best of our knowledge, no study in the literature has addressed this problem using the methodological steps investigated in this paper.

2. A deep literature review should be given, particularly advanced deep learning models in image processing. Therefore, the reviewer suggests discussing some related works by analyzing the following papers in the revised manuscript.

R: *The actual machine learning applications cover several areas in football data. Our literature review is basically focused on predictions, and some of these works are described in the Section Related Work in the paper.*
Lines 55-133 provide an overview of studies that investigate predictions in soccer context and Lines 294-302 describe how deep learning has been used to predict the tracking of players.

3. Please clarify the contributions to this field, for example, which are the existing ones, and which are your own ones?

R: *The contributions presented in our project have proven that it is possible to use complex network features to predict if the team with ball possession will reach the attack zone, i.e., will have chances of scoring.*

4. How about the computational complexity of the proposed method?
R: *The complete deep learning network architecture has 6.7 million of trainable parameters, where most of them (approximately 4 millions) belong to EfficientNet (the main backbone used in our formulation). Recall that those parameters (weights) are only fine turned in our trained strategy. Training the used architecture takes one day on a GPU NVIDIA (GTX 1050 Ti with 4 GB of RAM memory) with CPU (AMD FX-8350-e) and 16 GB of RAM memory. Recall that this machine has a very simple configuration, which demonstrates the advantage of using pre-trained models and simplifying our framework. This training procedure is performed off-line.*

The other components of the devised pipeline (e.g., construction of graphs, extraction of metrics from complex network, and the build of visual rhythms) take negligible time when compared to the training time of the network architecture.

5. Some future directions should be pointed out in the conclusion.
R: Thanks for pointing out this issue. In the current version of the manuscript, we extended the discussion upon possible research directions for future work. *Possible next steps include the implementation of graph-convolutional networks to improve the results and make the pipeline smaller. Another possible direction related to incorporating more spatial features related to the attacking and defending team, such as covered area and centroid of the team as Frencken and Lemmink work (*FRENCKEN et al., 2011*), We believe that the combination of complex network and spatial features may lead to improved prediction results.*

Reviewer 3

6. The Information highlighted, and the conceptual methodology, is already known and lots of advanced papers are already published.
R: *The paper aims to validate a new approach using concepts already consolidated, like graphs to analyze the players and teams behavior, visual rhythm in time series representation and machine learning to predict the play flow. It is possible that there are works using state-of-the-art algorithms, but our focus was to create and test the possibility of combination of graphs, time series representations and machine learning, in a unique pipeline with few data and heterogeneous teams. To the best of our knowledge, no study in the literature has investigated this combination for similar problems.*

7. No specific novelty is there.
R: *No algorithm, or metric was the focus of innovation during the development of this project, our attention was kept to create an innovation in the method of how to apply great tools available to predict the movement of the teams. In this paper, we aimed to create an effective framework that could lead to insights of how the attacking team may approach the penalty box, i.e., may have more chances of scoring. The main novelty of our work relies on the using of a complex network formulation perspective. Other papers analyzed teams using similar metrics to ours, but for different applications and objectives. The main contribution is to show that the use of complex network features to understand and predict the attack behavior is viable. Another contribution relies on the combination of time series representations with convolutional networks, pre-trained with images, to produce effective predictions. To the best of our knowledge, no study in the literature has addressed this problem using the methodological steps investigated in this paper.*

8. No System model and Algorithm is highlighted in the proposed approach.
R: *During the implementation, a neural network was utilized to make the predictions, the focus was to show how that makes it possible to create a pipeline that unify graph, time series, images and deep learning. It describes how a convolutional network is used and the amount of layers between lines 294 and 302.*

9. No Real Time case study based discussion.
R: *The idea to apply the concepts of this work is to consider the historical data of a time, create a tool to enable the understanding of how a team usually attacks and create a strategy to stop them. Real time application looks a great way to use this pipeline, but it can be a possible continuation of the work. Recall that a real-time application of machine learning algorithms on positional data is uncommon, despite its potential. We believe that is in part due to the enormous computational resources that might be needed.*

Reviewer 1

10. Case study: given the nature of the paper, a set of use cases with the relative Shap local explanation are required to provide the reader some results from the application of the proposed methodology. A bit of analytics would also improve the paper: who are the players mostly contributing with/without the ball to successful ball possession? What are the network features that define such players?

R: Thanks for raining such an issue, *Shap-based analysis may provide us the values for each channel of image (where each channel represents a metric). It is possible, therefore, to identify and appoint the most important players. However, in the development of the pipeline, this idea was discarded, because the training was realized with data of different teams. We plan to perform individual-based analyses in future work.*

11. Experiment replicability: authors provide both data and code of their work, this is a huge strong point compared with many other papers on the same topic. However, data description is missing, both within the paper and in the data repository. A broader data description would improve this aspect.
R: *Originally, the description of data was not made available. The data provided is now accompanied with metadata, including for example descriptions of labels and how information is presented in each column:* [*https://figshare.com/articles/dataset/REDSCAT2/19222746*](https://figshare.com/articles/dataset/REDSCAT2/19222746)
This organization leverages the replicability of our work and allows the validation of our results.

REFERENCES:

FRENCKEN, W.; LEMMINK, K.; DELLEMAN, N.; VISSCHER, C. Oscillations of centroid position and surface area of soccer teams in small-sided games. European Journal of Sport Science, Taylor & Francis, v. 11, n. 4, p. 215–223, 2011.
