# Supplementary material for: Using machine learning pipeline to predict entry into the attack zone in football
Source: PLoS One. 2023 Jan 18;18(1):e0265372. doi: 10.1371/journal.pone.0265372 (PMC9847968; doi:10.1371/journal.pone.0265372)

PLOS ONE

Response to Reviewers

Thank you for the opportunity to review our manuscript for the PLOS ONE. We appreciate the Reviewer's constructive comments and through reviews.

The content of the manuscript has been revised to address all the Reviewers' concerns. After addressing all the comments and revising the manuscript, we are submitting the revised version of our article for your consideration.

All changes made in the manuscript are highlighted. We considered all comments and notes carefully, and we are sure they represent a significant contribution to the quality of our manuscript.

We thank you for this opportunity. Please let us know if there are any further questions or concerns.

Sincerely

Authors

Title:

Using machine learning pipeline to predict entry into the attack zone in football

---

**Please note that all changes made to the manuscript, legends and figures are highlighted in yellow.

1. Please note that funding information should not appear in the Acknowledgments section/Funding section or Any other areas of your Manuscript. We will only publish funding information present in the Funding Statement section of the online submission form. Please remove any funding-related text from the manuscript.

R: *We have adjusted the Acknowledgements and removed all the funding information there.*

2. Please provide additional details regarding participant consent. In the Methods section, please ensure that you have specified (1) whether consent was informed and (2) what type you obtained (for instance, written or verbal). If your study included minors, state whether you obtained consent from parents or guardians. If the need for consent was waived by the ethics committee, please include this information.

R: *Our project was approved by the Research Ethics Committee of the State University of Campinas, protocol CAAE 56582616.8.0000.5404. And since all those present are of legal age, the need for consent was waived by the ethics committee, due to the video recording being done with proper cameras installed in the stadium in professional games. The Figure 1 shows how these cameras as installed.*

Figure 1: Cameras installed to capture the players during the match

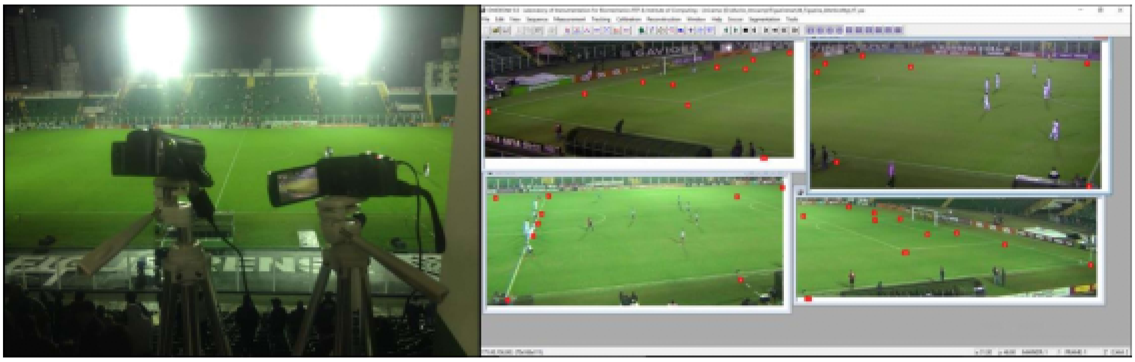

Supplement: Attachment — Submitted filename: PONE-D-22-05975R2_ResponseReview2.pdf [file pone.0265372.s004.pdf]
